# A Conceptual Analytics Model for an Outcome-Driven Quality Management Framework as Part of Professional Healthcare Education

**DOI:** 10.2196/mededu.4789

**Published:** 2015-10-06

**Authors:** Vasilis Hervatis, Alan Loe, Linda Barman, John O'Donoghue, Nabil Zary

**Affiliations:** ^1^ Department of Learning Informatics Management and Ethics Karolinska Institutet Stockholm Sweden; ^2^ Lee Kong Chian School of Medicine Nanyang Technological University Singapore Singapore; ^3^ Global eHealth Unit Department of Primary Care and Public Health Imperial College London United Kingdom; ^4^ Mohammed VI University of Health Sciences Casablanca Morocco

**Keywords:** Quality Management, Medical Education, Healthcare, Professional Education, Analytics, Decision Support Model, Computer-assisted Decision Making.

## Abstract

**Background:**

Preparing the future health care professional workforce in a changing world is a significant undertaking. Educators and other decision makers look to evidence-based knowledge to improve quality of education. Analytics, the use of data to generate insights and support decisions, have been applied successfully across numerous application domains. Health care professional education is one area where great potential is yet to be realized. Previous research of Academic and Learning analytics has mainly focused on technical issues. The focus of this study relates to its practical implementation in the setting of health care education.

**Objective:**

The aim of this study is to create a conceptual model for a deeper understanding of the synthesizing process, and transforming data into information to support educators’ decision making.

**Methods:**

A deductive case study approach was applied to develop the conceptual model.

**Results:**

The analytics loop works both in theory and in practice. The conceptual model encompasses the underlying data, the quality indicators, and decision support for educators.

**Conclusions:**

The model illustrates how a theory can be applied to a traditional data-driven analytics approach, and alongside the context- or need-driven analytics approach.

## Introduction

### Rapid Changes and the Implications for Educators

The quality of services offered by the health care organizations is closely associated with the quality of health care professional education [1]. Schools and educational programs prepare and train future health care professionals to become the next generation of caregivers. New health care professions emerge all the time [2], and new training programs must meet the needs of the evolving workforce. During their training, students are provided with the necessary theoretical knowledge, practical skills, and professional attitude.

Rapid changes within their specific domains cause the professional’s knowledge to differ from that of today’s students [3]. Scientific discoveries require new theoretical knowledge, technological development requires new skills, and new working concepts require new professional attitudes (eg, collaboration with multidisciplinary and international groups).

New pedagogical approaches (eg, flipped classroom, gamification, or simulation) that engage learners in their personal development by encouraging lifelong learning and increasing their skill set [4] have been tested, discussed, and evaluated, with promising results [5]. To continually redesign the curriculum is a real challenge for educators and educational developers [6]. The most demanding task is to choose appropriate teaching and learning activities. Therefore, when it comes to decision-making support, the development of novel methods becomes essential, especially when driven by educational purpose.

Analytics has successfully supported decisions in other areas (business, military, intelligence, politics, and economics). Though analytics we can interpret data and predict trends by calculating correlations [7].

### Related Work

Educational Informatics is a multidisciplinary research area that uses Information and Communication Technology (ICT) in education. It has many sub-disciplines, a number of which focus on learning or teaching (eg, simulation), and others that focus on administration of educational programs (eg, curriculum mapping and analytics). Within the area of analytics, it is possible to identify work focusing on the technical challenges (eg, educational data mining), the educational challenges (eg, Learning analytics), or the administrative challenges (eg, Academic- and Action analytics) [8].

The Academic- and Learning analytics fields emerged in early 2005. The major factors driving their development are technological, educational, and political. Development of the necessary techniques for data-driven analytics and decision support began in the early 20^th^century. Higher education institutions are collecting more data than ever before. However, most of these data are not used at all, or they are used for purposes other than addressing strategic questions. Educational institutions face bigger challenges than ever before, including increasing requirements for excellence, internationalization, the emergence of new sciences, new markets, and new educational forms. The potential benefits of analytics for applications such as resource optimization and automatization of multiple administrative functions (alerts, reports, and recommendations) have been described in the literature [9,10].

### Dimensions and Objectives of Academic- and Learning-Analytics

The field of analytics is multidisciplinary and involves different techniques, methods, and approaches. Some practitioners divide the performed actions into three different dimensions: time, level, and stakeholder. For each of these dimensions, specific analytic approaches may be applied to address specific questions.

In the dimension of time, Descriptive analytics produces reports, summaries, and models to answer: What, how, and why did something happen? Analytics monitors processes and provides real time alerts and recommendations to answer: What is happening now? Predictive analytics evaluates past actions and estimates the potential of future actions to answer: What are the trends, and what is likely to happen? Analytics also simulates the effects of alternative actions and supports decisions. Using analytics, choices are based on evidence rather than myths [11].

The scope of analytics can also be classified into five levels: course, department, institution, region, and national/international [12].

**Figure 1:**
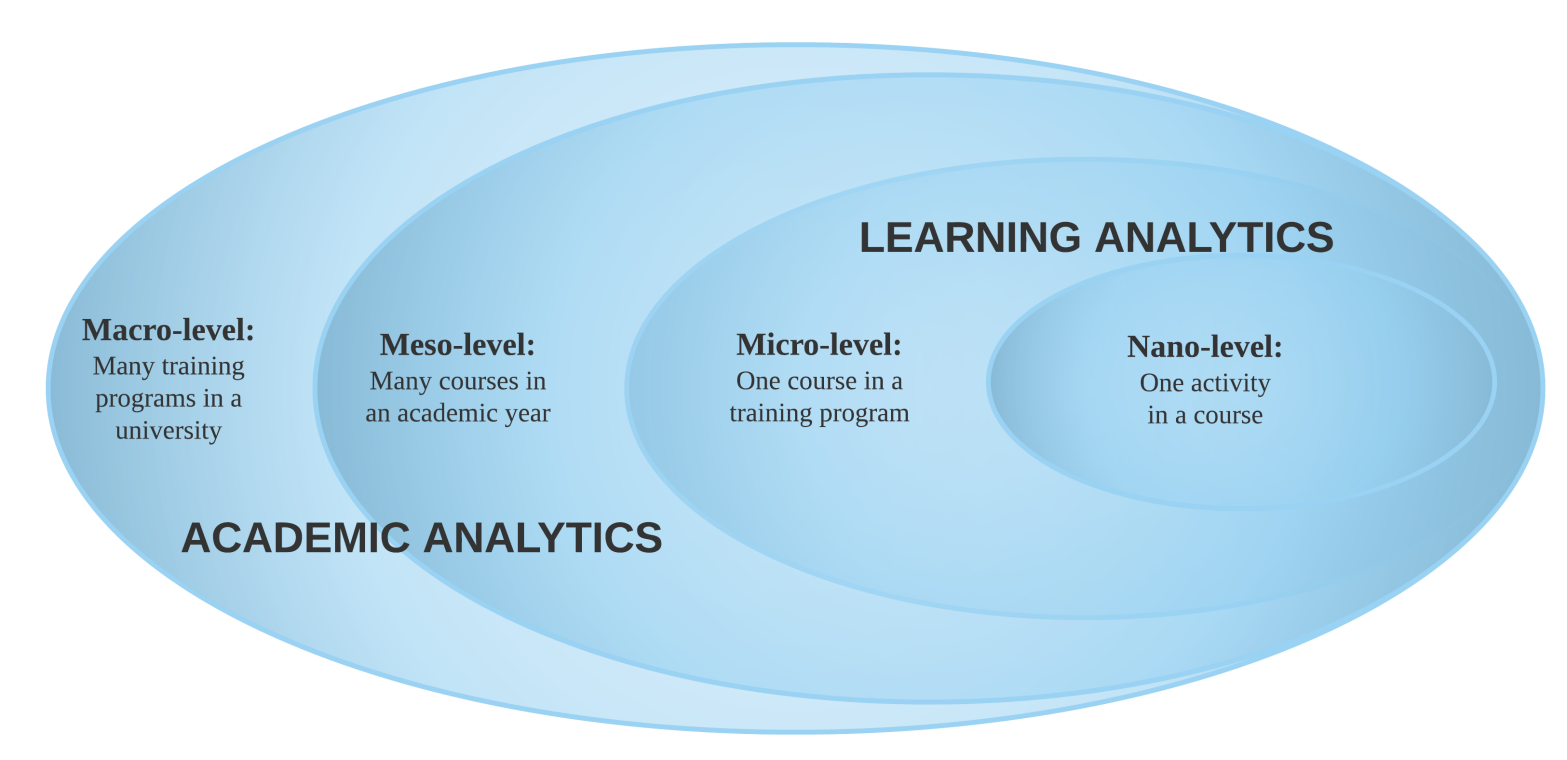
The curricular levels and overlaps of the different forms of analytics.

More specific terms have been used. The term “nano level” refers to one activity in a course, “micro level” refers to a course in a training program, “meso level” refers to many courses in an academic year, and “macro level” refers to many training programs in a university [13]. Figure 1 illustrates the relationships between these levels.

The term “Learning analytics” refers to operations at the micro and nano level, when the focus is on decisions concerning achievements of specific learning outcomes. “Academic analytics” applies to the macro and meso levels, when the focus is on decision making regarding procedures, management, and matters of operational nature [14]. Figure 1 illustrates how the different forms of analytics overlap.

The application of analytics can also be oriented toward different stakeholders, including students, teachers, administrators, institutions, and researchers. They may have different objectives, such as: mentoring, monitoring, analysis, prediction, assessment, feedback, personalization, recommendation, and decision support. Analytics can apply different techniques, including visualization, data mining (including classification, clustering, and association), and network analysis within those different tasks.

Chatti et al [15] propose different dimensions: The environment; what data is available? The stakeholders; who is targeted? The objectives; why do the analysis? And the method; how has the analysis been performed [15]?

### Introduction of the Underline Theory and the “Analytics Loop”

Even if the dimensions or the methods and objectives of the approaches described above differ, the fundamental idea of the five steps [16] of the analytics loop is always the same. The five steps of analytics are:

1. Capture: Access to data is the foundation of all analytics. This data can be produced by different systems and stored in multiple databases. One great challenge for analytics projects in this step is that necessary data may be missing, stored in multiple formats, or hidden in shadow systems.

2. Report: This step involves creating an overview to scan. Different tools can be used to create queries, examine information, and identify trends and patterns. Descriptive statistics and dashboards can be used to graphically visualize eventual correlations.

3. Predict: Different tools can be used to apply predictive models. Typically these models are based on statistical regression. Different regression techniques are available and each one has limitations.

4. Act: The goal of any analytics project is to provide information based on predictions and probabilities that support decision making. Analytics can be used to evaluate past actions and estimate the effects of future actions. In that way, analytics can provide alternative actions and simulate the consequences of different actions.

5. Refine: This is the self-improvement process. The monitoring, feedback, and evaluation of the project’s impact create new data and evidence that can be used to start the loop again with improved performance [16].

### Aim and Objective for This Study

The aim of this study is to investigate how analytics could contribute to the quality management and improvement of health care professional education. We aim to provide a deeper understanding of the process of synthesizing the available educational data to create the necessary information to support decisions. The research question we seek an answer to is: How can the different parts of the analytics loop (Capture, Report, Predict, Act, Refine) after Campbell et al [16] be described in a conceptual model for an analytics-driven quality management framework in health care professional education?

## Methods

### Study Design

Every research project consists of five key components: goals, theoretical framework, research questions, methods, and validity [17]. A number of other factors can also affect the design and thus make all empirical research unique. Traditional research follows a linear approach. This qualitative study follows an interactive approach [18]. According to this approach, interconnections between the components and the structure are flexible, and the research questions are not the starting point. Instead they are at the center, like a hub, and they connect directly to all of the other components [18].

We expand, one by one, the five key components (goals, theoretical framework, research questions, methods, and validity) [18] in order to clarify the chosen design.

There are three goals in conducting this study: personal, practical, and intellectual. The personal goals which motivated us to do this study include curiosity about the specific theory and a desire to change the existing situation. The practical goals focus on finalizing a conceptual model to describe how the theory works. The intellectual goals are to understand the processes, identify eventual gaps, and provide explanations.

The theoretical framework is based on the fact that analytics has been successfully used in other areas. Academic analytics has been defined as the application of business intelligence in education [19]. The value of analytics for higher education has been described as very positive. Analytics in general could generate insights based on data and improve the decision making process [12]. Learning analytics could support decisions and improve learning, and Academic analytics could make the quality management of educational procedures easier and more effective.

The theory of the analytics loop is well described, but we have yet to see its full potential implemented in an analytics-driven quality management process in the setting of health care professional education. The research question thus focuses on providing deeper understanding, insights, and explanations. The main research question could be divided into three sub-questions: (1) How does the quality improvement work in health care professional education today? (2) How could this process be changed using academic analytics, and (3) What inputs are necessary to get the theory to work in practice.

The overall methodology follows the deductive case-study approach [20] and develops a conceptual model [21]. The design was deduced to test the theory of the analytics loop (capture, report, predict, act, refine; after [19]). The conceptual framework has been used to explain key factors in graphical form.

### Strengths and Weaknesses

Case studies can be used to illustrate real problems and needs and help us to identify gaps in processes. A clear limitation is the potential to generate recommendations, solutions, or generalizations recognized as normal in other contexts.

The case study method includes interviews and observations. Responders included five teachers, two course designers, two directors, and two coordinators during a review to improve the quality of a masters’ program in health informatics. Analysis of the text generated by the responders has been re-used to create the conceptual model. The case in this study is considered a middle-step to help us understand the needs and illustrate the gaps.

The main focus is on the creation of a conceptual model to test the theory in general. The combination of the methods allows us to close the loop and validate the outcomes.

### Validation

The validation and empirical implementation of the study includes three steps: (1) mapping of the actual quality improvement process, (2) development of a case to illustrate eventual benefits of Academic analytics, and (3) creation of a conceptual model to identify the needed data.

### The Case Study

A teacher suspects gaps in the curriculum of a course and wants to improve its quality by adding a new type of teaching activity. What is the current practice, and how would this scenario change after using analytics?

## Results

The first two sections summarize observations from the responders’ interviews, and describe challenges and the current quality improvement processes. The third section summarizes how the different analytics solutions can be used in this particular case. The last section describes how the theory works.

### The Challenges and the Request of Constant Improvements

The educational program managers and faculty face two kinds of challenges which require constant curricular changes and improvements.

The first challenge pertains to the course content. The syllabus is influenced by recommendations of the International Medical Informatics Association [22]. However, the field of health informatics is continuously changing and growing; new techniques, aspects, theories, and solutions emerge constantly, and teachers have to renew the teaching material and course curricula annually. During this reform, according to DaRosa & Bell [6], we consider the curriculum to be a product. This improvement is time-consuming but relatively easy. The faculty are familiar with the new knowledge; they have time to prepare the material and the examination type is easy to define. Eventual success or failure is easy to measure.

The second, and greater challenge, is to improve and adapt the methods to provide this content. According to DaRosa & Bell [6] the curriculum is now a process. The focus is to engage the students to be active participants and not passive recipients of prescribed knowledge. The students in this masters’ program are already practitioners in their field; either computer engineers or health care professionals. The program instructors use different teaching approaches (case projects, group-work, team-based learning) rooted in different learning theories, like constructivism, experiential-based learning, and connectivism. They constantly try to improve the course, which proves to be demanding work. The achievements or objectives are more generic in nature (problem-solving ability, engagement in personal learning, critical thinking), the examination is sometimes unclear, and there are no pre-defined measurements of success or failure.

### The Current Quality Improvement Process in Different Levels

During course development, coordinators have to define, re-define, or improve the course content; choose appropriate teaching activities; and follow policies and recommendations. During this phase, they work in close collaboration with other teachers, instructors, course designers, and program managers, and they have access to other experts from the department. Some instructors are willing to test and implement new pedagogical approaches in their sections, like the flipped classroom approach, gamification, or simulation. They can always share ideas and experiences and get feedback, but they admit that sometimes they rely only on a “gut feeling,” especially in the case of new implementations. While the course is in session, the coordinators again have to trust this “gut feeling” in the hope that everything will turn out well.

The students give their feedback through a Web-survey after the course ends. The feedback pertains to the alignment between the course objectives, teaching activities, and supporting learning materials, as well as with other courses in the program, and communication and collaboration with other students and teachers.

The course coordinators compile and send a final report of the survey results to the program management. This report includes the coordinators’ own reflection of the results and plans for future changes and improvements.

The program director, students, and teachers convene once a year to conduct a SWOT-analysis (Strengths, Weaknesses, Opportunities, and Threats), which leads to an action plan for the following year. The program director introduces this action plan to the program committee and the board of education.

The board of education closes the loop by sending feedback to the program director and faculty, which the course coordinators consider during course development.

### How Analytics Can Change This Scenario

In this scenario, we can identify all the different dimensions described (in section 1.2). Before the course starts and during the preparation stage, the course coordinators can use curriculum mapping tools to identify the gaps precisely. They can get insight into what kind of learning objectives are not properly supported by teaching or learning activities. They need suggestions for new appropriate and motivational teaching activities to add into the schedule. Furthermore, through other analytics tools, they can analyze the class and predict their needs. Examples include student demographics, previous performance, approaches to learning, the blend of technology used, and group dynamics. This kind of data can be processed by a range of algorithms and predictive models to develop the probable characteristics of the class [23].

In the next step, other visualization tools can provide alternative suggestions for designing appropriate activities to fit this particular class and demonstrate the effects of each. The coordinator can monitor the activities and progress during the ongoing course. They can zoom in and out from the entire class to one working group or one student. They can also follow the progress of the generated social networks. They can calculate the general engagement and identify students at risk. In an open platform, they can also compare current analytics from this class with other, anonymized datasets within the same educational program, from other faculty, or even from similar courses in other universities [23].

The results and experiences generated by the course can be used to build up the knowledge database with evidence about different pedagogical interventions. This can assist in contextualizing the Policy and Environmental analytics within the organization (department or university) and be a key part of the quality improvement and academic research.

### How the Theory Can Work With the Existing Data

#### Analytics Loop Step 1: Capture

**Figure 2:**
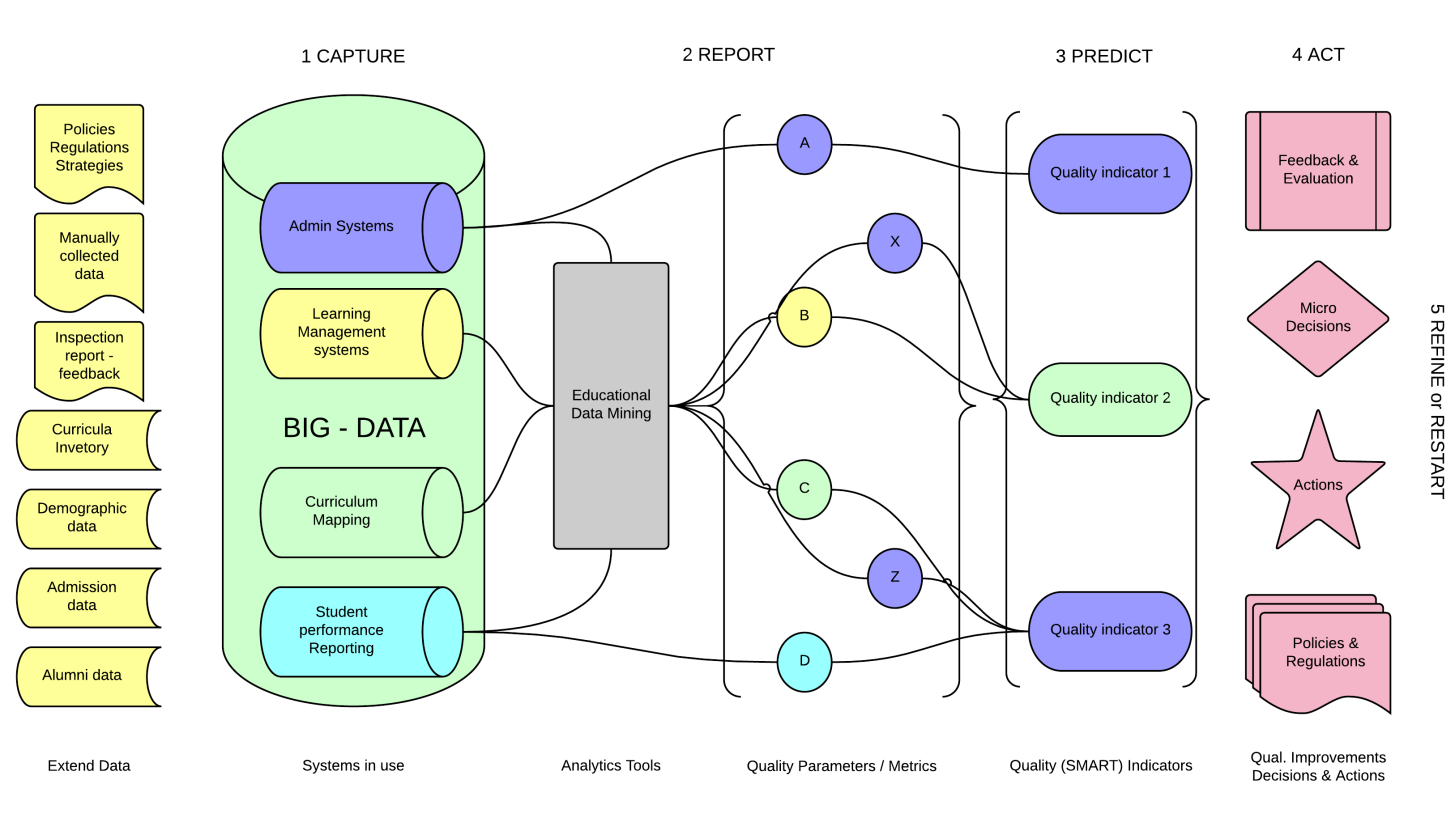
Analytics-driven quality management framework.

We had access to a collection of large and complex datasets which we will call “big-data”. In this context, “big” refers to complexity rather than volume. Examples of big-data include: demographics, admissions, syllabuses, curricula, course evaluations, teacher’s reflections, students’ performances, and logs (see column 1, Figure 2). The included systems collect and store more data on a daily basis. Examples of systems are course administrative systems, learning management systems, curriculum mapping systems, and student performance reporting systems (see column 2, Figure 2).

#### Analytics Loop Step 2: Report

Different types of data are collected: numeric, text, statistics, structured, metrics, and values. Using different analytics tools [24,25], the data is processed (see column 3, in Figure 2) and transformed into metrics or parameters (eg, Boolean operators: True/False (see column 4, Figure 2)).

#### Analytics Loop Step 3: Predict

The challenge here is not lack of data, but synthesis and interpretation. Other analytics research projects have focused on data collection and its processes (eg, tracking mobile data and the use of standards [26]), or on data processing techniques (eg, data mining [27], and better reporting or presentation (eg, Visual analytics [28]).

Our focus is on the synthesis and decision support. This synthesis includes three main steps: The creation of SMART (Specific, Measurable, Achievable, Relevant, and Time-bound) quality indicators, transformation of the analyzed data into meaningful information, and presentation of the correlation between the nodes (column 5, in Figure 2).

The creation of quality indicators is the key step in this model. Some indicators are simple (eg, more than 75% of students have done one exercise before the deadline). It’s easy to identify the supported data and easy to check. However other indicators are complex (eg, the students are engaged in their studies, the students are developing a professional attitude, or the course is linked to relevant research.)

The next step is identifying the necessary parameters or metrics. The analytics tools (see column 3, Figure 2) process all captured data and all parameters/metrics and eventual correlations between them (see column 4, Figure 2). The challenge is to select the most important of these. The importance or need for specific entities depends on the quality indicator which can change from case to case. The context (eg, the class size or education level) determines whether a metric is relevant.

The last step before making a recommendation can be done by visualizing or highlighting correlations or causalities (or lack thereof) between the chosen metrics.

The most important factor in this data transformation is first to inform and further to support decisions by setting a clear and specific context. Data or metrics, correlations between them, and eventual conclusions and recommendations are relevant or not depending on the context.

Analytics Loop Step 4: Act

The goal of any analytics project is actions based on data-supported decisions. The decision-making process however is sensitive and complex (see column 6, Figure 2). Even if the evidence, analysis, and data are enough to convince and support one decision, the decision makers may act differently. This inconsistency depends on a number of factors, including context, needs, economy, resources, climate, policies, ecosystem, and circumstances.

Analytics Loop Step 5: Refine

Evaluation of the analytics process can provide valuable feedback. This new data and evidence can lead to correction and modification of the analytics loop. Evaluation of the implemented actions and interventions create new data, which can lead to better suggestions and recommendations.

##  Discussion

### Principal Findings

This study develops a conceptual framework to describe how analytics can support the decision making process and improve the quality of health care professional education. The starting point was to follow the steps of the analytics loop, to examine how it works and illustrate the challenges. We can read this model in both directions.

### Data-Driven Analytics Approach

**Figure 3:**
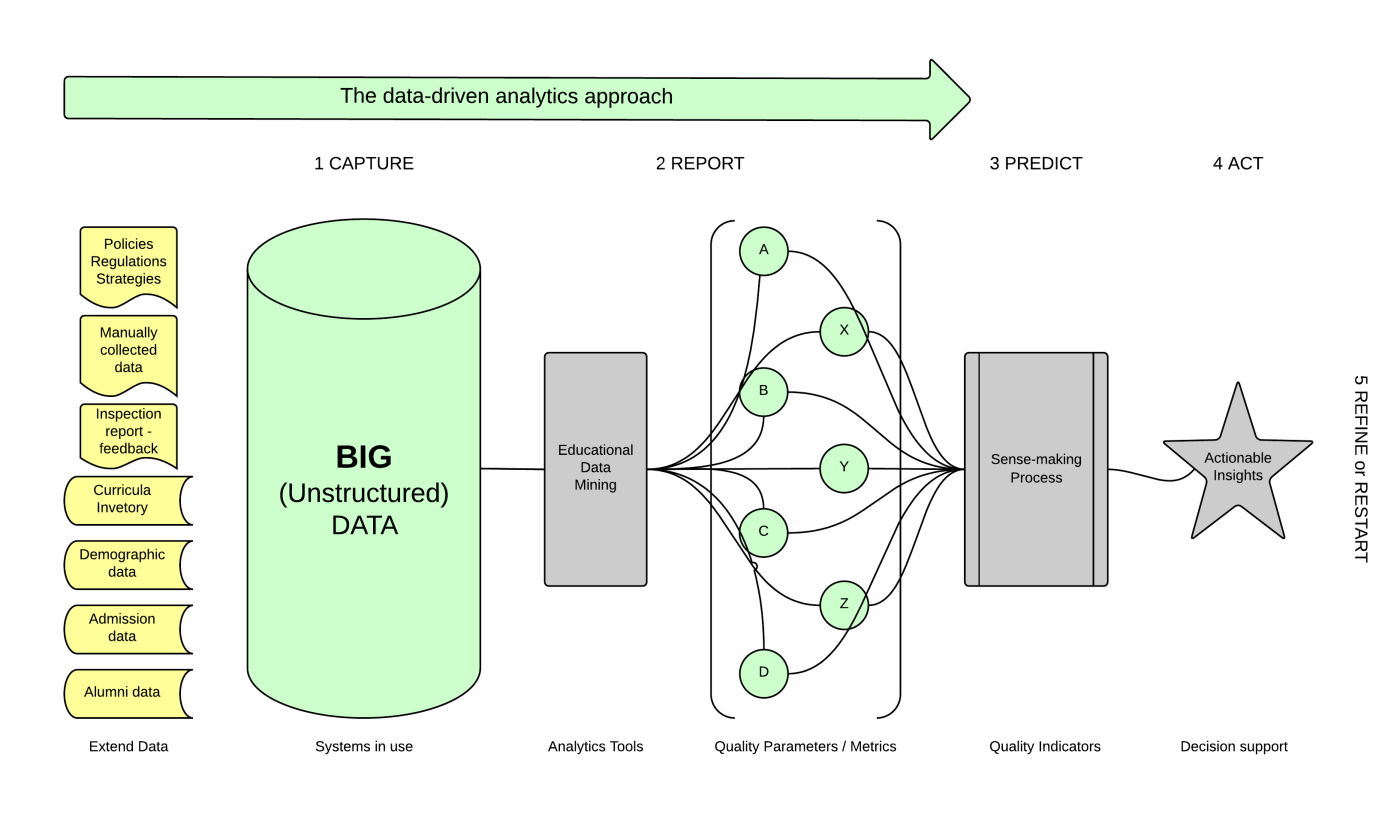
The data-driven analytics approach.

Reading from the left to the right, the framework describes the common and traditional data-driven analytics approach, which is more meaningful to experts in the data-mining area. It starts from the data and ends at the decision. According to this approach, we start the loop capturing as much data as possible and then push it through the steps. The main focus is on the data and the techniques to collect, store, clean, secure, transfer, process, and access the data. In the next step of reporting, a large amount of data is an asset. The more data we put in, the better reports we get out. However, processing such large datasets also presents a challenge, requiring more advanced mining techniques and more powerful computers, tools, and software. To make sense of all this data, calculate all trends, and investigate all possible correlations is a demanding task. Educational data-mining (see column 3, Figure 3) is clear just to experts in the data-mining area, a drawback of the approach. Results from the analytics engine might be accurate, precise, and based on evidence, but still remain just suggestions. Sometimes the decision makers, because of unknown circumstances, don’t accept the recommendations and act differently. In this data-driven approach, decision support (see column 6, Figure 3) is like a black box, and the processes within it are unknown. According to the analytics loop, the last step is to start all over again with more data.

### Context- or Need-Driven Analytics Approach

**Figure 4:**
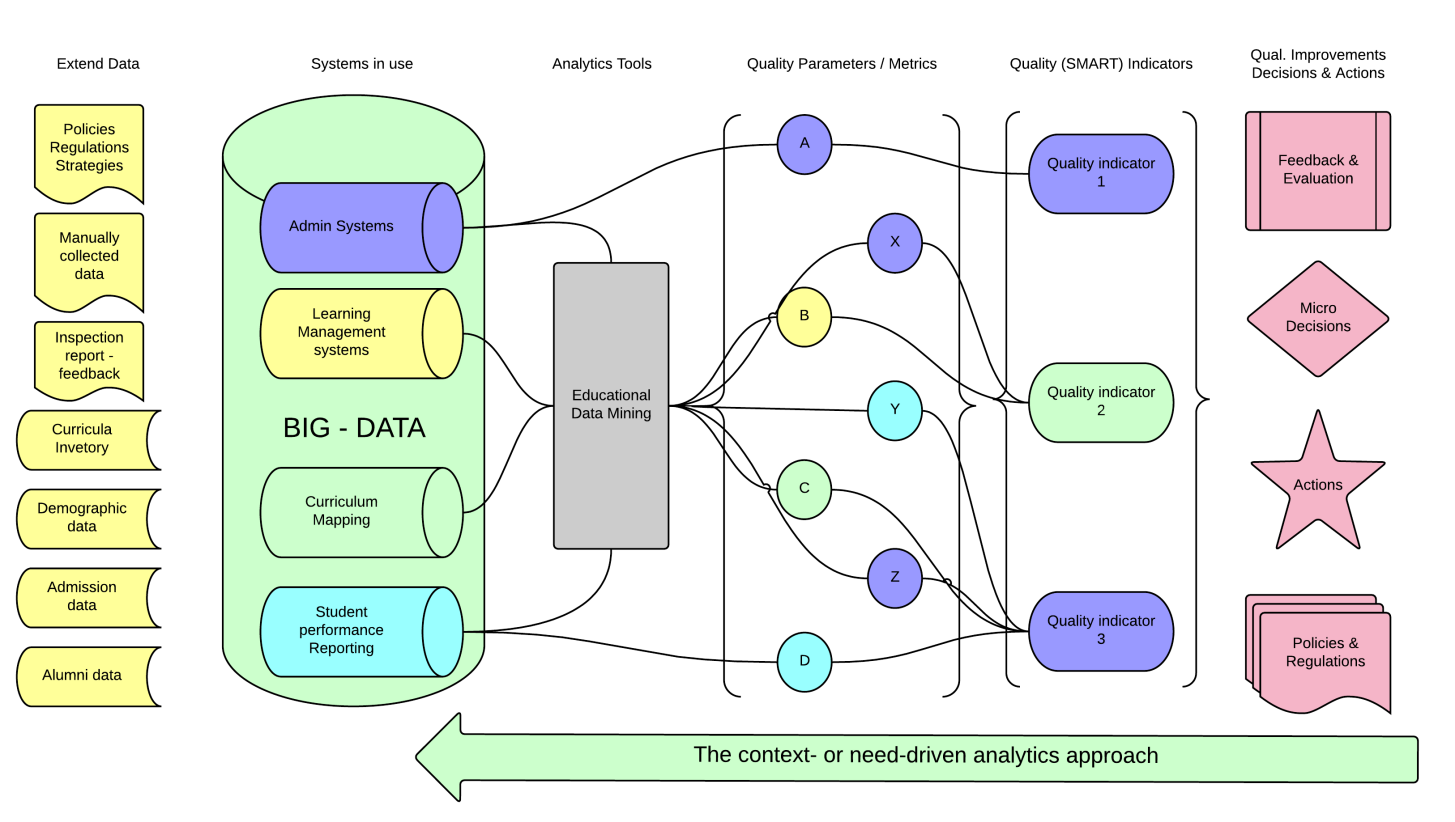
The Context- or need-driven analytics approach.

We can also read the model from the right to the left. The framework describes a new analytics approach that we can call context- or need-driven analytics. This approach is more suitable for non-technical oriented educators and decision makers (like faculty in health care professional education). The approach starts from the need for a decision, and goes through analysis of relevant data. Quality improvements, decisions, and actions (see column 6, Figure 4) must be crystal clear. All details are important: the stakeholders, the context, special needs, financial limits, access to resources, organizational climate, existing policies, technological ecosystem, timing, and other circumstances which affect the decisions. The outcomes from this analysis are the request of specific information to support a decision or micro-decisions. This relevant and specific information results from synthesis of carefully selected and specific data. These data are selected, processed, calculated, compared, mined, and operated by analytics tools using specific mining techniques. The analytics engine contains other integrated mechanisms and specialized agents to identify systems producing the data or repositories containing the data. This time we pull the data and processes from educational data mining (see column 3, Figure 4), which is like a black box for this group of users. The last steps of the analytics loop either refine the data to answer the primary question, or generate a more specific question to restart the analytics process.

### Data-Driven Approach Versus Context- or Need-Driven Approach

Big data alone, even lots of it, may become cumbersome and demanding to manipulate, hence weighing down processes intended to achieve beneficial results.

Although effort and time can be conserved at the onset of adopting a context- or need-driven approach (ie, by eliminating options deemed unsuitable based on contextual standpoints), the very nature of this approach demands specifics. This presents the likelihood of missing possible underlying problems overlooked by the user, which surfaced in trends detected from optimized data-driven strategic approaches.

The context- or need-driven approach can serve to identify specific areas of concern, but it cannot function with a dearth of big data. Optimized data-driven strategic approaches can help drive root-cause analysis to identify all possible interactions within the system that could contribute to areas of concern.

### Analysing the Use Case From the Context- or Need-Driven Approach

The scenario of the use case is the same as before; a course coordinator identifies a gap in the curriculum and asks for support to choose proper learning activities for this group. The coordinator starts from column 6 in Figure 4 and takes into account all the circumstances (context, special needs, financial limits, available resources, existing policies and organizational climate, technological ecosystem, timing, and all other parameters) that can affect the decisions. After this primary analysis, some suggestions would be excluded to save effort and time (eg, activities for which we don’t have money to buy equipment). The creation of clear and concrete quality indicators in the next step (see column 5, Figure 4) is important, especially when some indicators are compound and complex. Quality indicators include: which of the suggested teaching activities engage the students in their studies, and which activities help students to develop a professional attitude.

Column 4 in Figure 4 contains specific and carefully selected metrics or parameters (eg, numeric data or Boolean operators: True/False) that are necessary and directly connected to the quality indicators.

The biggest challenge in this approach is the development of tools, mechanisms, operators, and agents that can recognize, select, pull, and process the relevant data to support the creation of those parameters (see column 3, Figure 4).

Columns 1 and 2 are the same as before, but indexing, structuring, and standardizing all data before storing it could facilitate the process.

### Knowledge Transfer From Other Areas

Supporting decisions by data and evidence has been used in other application domains in the past. Some Clinical Decision Support Systems (CDSS) use these principles (ie, comparing specific selected parameters from one individual patient against standards). Medical doctors and other health care professionals use this kind of (CDSS) systems to determine a diagnosis or choose a treatment.

Using the same metaphor as the previous use case, teachers and course designers can check the qualitative health of a course, identify problems, suggest appropriate treatment, and monitor the progress. We can observe some characteristics of the CDSS and learn some lessons.

These systems are extremely precise but also highly specific and specialized for just one or a limited number of purpose(s). Universally applicable systems which can diagnose all possible diseases do not exist.

Such systems access and process only specific data sets, leaving out redundant ones.

The essential power of these systems is to clarify incomprehensible data sets, and in this way, support decisions.

### The Scientific Value and the Practical Use of This Study

One challenge when trying to connect analytics and education is to communicate issues and theories concerning one area to specialists and experts from the other. Case studies and conceptual models have been used successfully for this purpose. In general, a case allows us to apply an idea or theory to a particular context and to test it from different points of view.

The case we have used illustrates a real problem. A clear limitation of using the case studies as a method is the ability to make generalizations. The challenges and process are specific only for the involved personnel. Any recommendations and solutions for the particular case do not apply in other contexts. We used the case to understand the practical use of analytics and illustrate the communication problems between experts from the areas of analytics and education.

The conceptual model demonstrates how the theory of the analytics loop can be applied to both the data-driven analytics approach and the context- or need-driven analytics approach with respect to each groups’ terms and conditions. The model also identifies the challenges. A different approach might result in different recommendations.

## Conclusions

All parts of the analytics loop [19] can be conceptualized both in theory and practice. Decisions that improve the quality of education can be driven by synthesizing existing data rather than acquiring more data. Data can be transformed to information when it has been contextualized. Analytics projects do not always have to follow the traditional linear data-driven approach. The context has high impact on the decision making process. The context- or need-driven analytics approach emphasizes inclusion of all necessary details and is more suitable for non-technically oriented users.

Beyond recognizing the promise and potential of new technologies, mapping how ICT (analytics) and ICT-based learning (education) fit in the design and implementation of health care professional’s education remains a vital task which, as seen from Figure 2, lies within the middle columns, away from the extremities.

Hence, in the best interests of both the students and the organization, these two approaches should ideally co-exist and function in tandem. Instead of dichotomizing our choices, we should explore the middle ground.
